# Variable responses to tree root exclusion by understory plant functional types in a xeric longleaf pine woodland

**DOI:** 10.1093/aobpla/plag032

**Published:** 2026-07-18

**Authors:** Phoebe A Judge, Ricardo M Holdo, O Stribling Stuber, Steven T Brantley

**Affiliations:** The Jones Center at Ichauway, 3988 Jones Center Drive, Newton, GA 39870, United States; Odum School of Ecology, University of Georgia, 140 E. Green St., Athens, GA 30602, United States; Odum School of Ecology, University of Georgia, 140 E. Green St., Athens, GA 30602, United States; The Jones Center at Ichauway, 3988 Jones Center Drive, Newton, GA 39870, United States; The Jones Center at Ichauway, 3988 Jones Center Drive, Newton, GA 39870, United States

**Keywords:** C_4_ grass, competition, facilitation, forb, gas exchange, leaf water potential, legume, soil moisture, stomatal conductance

## Abstract

Plant species interactions in woodlands help maintain the coexistence of trees and groundcover, contributing to high plant diversity. Interactions can be either positive or negative and take place both above and below ground. Both competitive and facilitative interactions have been documented in longleaf pine woodlands, but the dynamics of these interactions and their effects on plant physiology are poorly understood. Our objective was to quantify the impact of tree root exclusion on physiology and growth of understory plant functional types (PFTs) that represent the diversity of longleaf pine woodlands. We used trenching to isolate understory plants from tree roots and compared soil moisture, leaf water potential (Ψ), leaf-level gas exchange, and plant growth for four understory species representing different PFTs in trenched root exclusion plots and untrenched control plots. We hypothesized that root exclusion would reduce understory plant performance compared to untrenched control plots by isolating plants from facilitative effects of canopy trees, but our hypothesis was not supported. We observed better plant performance in trenched plots for some PFTs, and no differences in plant growth for any PFT. These data suggest competitive effects of tree root presence, but results varied among PFT. The study period was unusually wet, with rainfall 53% above normal. In the context of previous studies, these results suggest that belowground competition is more important than facilitation during a wet year, and the relationship between longleaf pines and understory plants shifts from facilitation to competition depending on rainfall.

## Teaser

This study examined interactions between tree roots and understory plants. Contrary to expectations, excluding tree roots did not reduce understory plant performance or growth. Some understory plant groups actually performed better when tree roots were excluded, suggesting that belowground competition dominated interactions during this study. However, because the study occurred during an unusually wet year, these findings indicate that interactions between trees and understory plants can shift between competition and facilitation depending on rainfall.

## Introduction

Savannas and woodlands are known for having high groundcover diversity, which supports a variety of ecosystem services (Marchant 2010, Williams et al. 2022). While disturbances such as fire promote the coexistence of trees and grasses, biotic interactions also affect plant diversity (Scholes and Archer 1997, Ward et al. 2013). Many studies have focused on belowground competition for resources, especially water, as a primary driver of community structure (Nippert and Knapp 2007, Cramer et al. 2012, Ward et al. 2013), while other studies have focused on aboveground competition because many grassland species are shade-intolerant, and intense competition for light can reduce understory density (Vadigi and Ward 2013, Kirkman et al. 2016, Brantley et al. 2024). Comparatively few studies have examined the role of facilitation in shaping open woodland plant diversity, but recent evidence suggests that facilitation—defined here as an interspecific interaction in which one or both plants benefit in terms of growth, survival, and/or reproduction—is a key process in these ecosystems (Synodinos et al. 2015, Loudermilk et al. 2016, Oliveira-Neto et al. 2022). For example, large trees can be particularly important in mediating the coexistence of species at small scales through both above- and belowground interactions such as shading or hydraulic redistribution, which tend to buffer environmental extremes (Loudermilk et al. 2016, Barron-Gafford et al. 2021, Oliveira-Neto et al. 2022). While competition for water and nutrients can be intense (Ward et al. 2013, Barron-Gafford et al. 2017), processes like hydraulic lift can benefit understory species through facilitation, potentially affecting groundcover diversity (Barron-Gafford et al. 2021, Belovitch et al. 2024). While many savannas and woodlands are dominated by drought-tolerant C_4_ grasses, other plant functional types (PFTs) are also abundant and represent the majority of species in these systems, and these species are potentially more vulnerable to worsening droughts (Kirkman et al. 2016). The strength of tree–groundcover interactions often varies across PFTs with more drought-tolerant species, e.g. C_4_ grasses, being better able to tolerate competition for water and nutrients, while coexisting forbs and woody seedlings are more dependent on positive interactions (Vadigi and Ward 2013).

Belowground effects of trees on understory plants are largely mediated by the effects of tree roots on resource availability, whether from competition, facilitation, or both. Longleaf pine woodlands are an ideal ecosystem to test the effects of belowground interactions between roots, but relatively little such research has been done in this ecosystem. Longleaf pine ecosystems support a continuous layer of diverse groundcover plants representing a wide variety of PFTs, including drought-tolerant C_4_ grasses, legumes, and numerous forbs, including over 120 rare and endangered plants that could be susceptible to climate change (Noss 1989, Peet and Allard 1993, Walker 1993). The longleaf pine ecosystem once dominated much of the southeastern US Coastal Plain, but timber harvesting for turpentine and lumber, conversion to agriculture, fire suppression, and soil disturbance have reduced the extent of longleaf pine forest to ∼5% of its former area, and only a portion of this remaining area has intact understory vegetation (Peet and Allard 1993, Simberloff 1993, Cox et al. 2004). Longleaf pine ecosystems and the understory communities they support could be under further threat from climate change due to declining summer precipitation, increased occurrence of severe flash droughts, higher evapotranspiration rates, and increasing soil moisture deficits (Kunkel et al. 2013, Zhao and Dai 2015, McNulty et al. 2019).

Both facilitation and competition can be important in shaping understory plant communities in longleaf pine ecosystems (Loudermilk et al. 2016, Belovitch et al. 2024). While some studies have shown strong negative effects of midstory oaks on groundcover and longleaf regeneration (Brantley et al. 2024, Loudermilk et al. (2016) showed that midstory oaks enhanced survival of <2-year-old longleaf seedlings by reducing seedling moisture stress. They further suggested that competition from oaks was not an impediment to seedling survival and that the facilitative effects of oaks via soil surface temperature outweighed any negative interactions from competition (Loudermilk et al. 2016). Other studies have tested the effects of trenching to exclude overstory tree roots on understory plants. For example, Pecot et al. (2007) found that trenching to exclude roots in longleaf pine increased understory hardwood growth, and this was likely due to reduced competition for belowground resources. Finally, a field experiment at the same site used in the present study deployed trenching to sever roots and test for water stress effects on understory plants (Belovitch et al. 2024). The study found that trenching limited access to hydraulically lifted water (HLW), and understory plants in trenched plots experienced significantly lower predawn leaf water potentials (Ψ) during the latter half of the growing season compared with conspecifics in control plots.

A better understanding of how tree root presence or absence affects understory plant physiology and growth will help us predict how tree density influences the PFTs of understory communities under future climate conditions. Thus, our objective was to determine the effect of tree root presence/absence on the physiology and growth of four herbaceous understory species representing four different PFTs in xeric longleaf pine woodlands by experimentally excluding tree roots through trenching. Prior studies at the site suggested that understory plants where tree roots had been excluded showed more water stress than untrenched control plots due to access to HLW in the control treatments (Belovitch et al. 2024). Therefore, we hypothesized that soil moisture would be lower in root exclusion plots and that the plants in these plots would have more negative leaf water potential, lower stomatal conductance, and lower photosynthetic rates, compared to plots where roots were present. We also compared growth and biomass parameters of understory herbaceous plants in plots with and without tree roots. We hypothesized that plants in untrenched control plots would have greater individual growth rates and biomass than plants in trenched root exclusion plots. Furthermore, we hypothesized that the strength of these effects would vary among PFTs due to differences in the photosynthetic mechanisms represented in the groundcover community. These results will add to our understanding of the relative importance of belowground competition and facilitation for understory plant physiology and growth in pine woodland ecosystems.

## Methods

### Study site and experimental setup

The study took place at the Jones Center at Ichauway, an 11,400-ha private preserve located in southwestern Georgia, USA. This region is characterized by hot summers and no dry season (Peet 2006). Mean annual temperature at the site is 19°C, with average daily highs reaching 34°C in summer and mean annual rainfall is 1260 mm (Goebel et al. 2001, S. Brantley, unpublished data). The study was conducted on a xeric upland sand ridge characterized by deep, dry, sandy, and excessively drained soils in the Lakeland and Lucy soil series. As a result, the vegetation at this site is typical of a sub-xeric sandy upland community as characterized by Peet (2006), with a sparse woodland overstory dominated by longleaf pine (*Pinus palustris*) and drought-tolerant oak species such as turkey oak (*Quercus laevis*) and sand post oak (*Quercus margaretta*). Canopy leaf area index at this site was ∼1.8 and basal area ∼10 cm^2^ m^2^ (S. Brantley, unpublished data). The understory is also sparse compared to nearby sites, and bare soil patches are common. Understory vegetation cover is dominated by wiregrass (*Aristida beyrichiana*) and other warm-season grasses, but forbs and subshrub form oaks are common as well. The site is burned on a 2-year rotation, and the last prescribed burn at the study site occurred in February 2021, ∼3 months before the start of this study.

This stand was also the site of a previous study in which substantial Hydraulic Laift (HL) by longleaf pines and uptake of HLW by understory plants has been previously documented (Belovitch et al. 2022, 2024). Tree rooting depth has not been measured directly at this site, but a previous attempt to extract roots from a mature longleaf pine reached 5–6 m deep before the effort was abandoned (S. Taylor, personal communication). Isotopic data from two previous studies at this site also suggest that trees there can reach saturated soil layers (Ford 2004, Belovitch et al. 2022, 2024).

**Figure 1:**
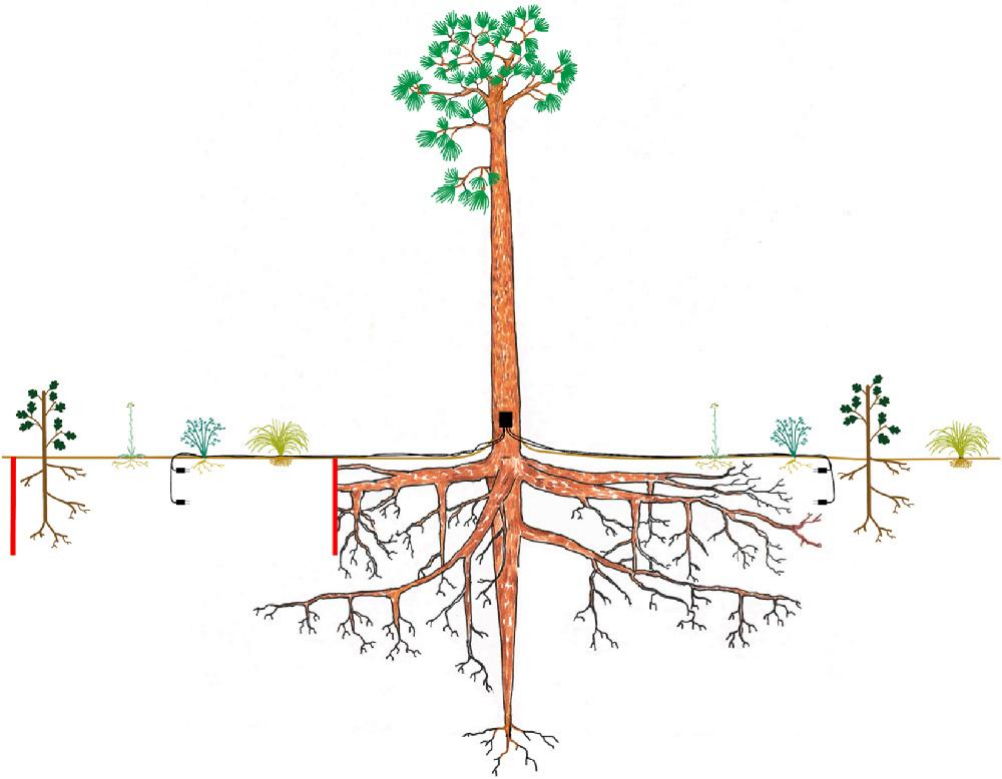
Example setup for an experimental block, showing trenched root exclusion plots (left) and untrenched control (right) plots, an individual plant from each functional type studied, and soil moisture sensors and data logger (mounted to tree). Vertical red bars represent trenches.

In April 2021, we randomly selected four longleaf pine trees from a pool of suitable candidates in the area. We chose longleaf pine specifically because it was the dominant canopy species at this site (>80% of basal area), it had shown evidence of greater hydraulic lift that co-occurring oaks in the previous studies mentioned above, and there is specific interest in restoring this species in southeastern USA. Suitability of the individual trees we selected was based on size (we focused on mature canopy trees), distance from neighbouring trees, lack of groundcover disturbance, and similarity in understory composition and density. Tree sizes ranged between 40 and 55 cm diameter at breast height. Trees were 30–160 m apart, so all four trees were located within ∼1 ha area. The minimum distance reflected typical spacing of mature trees in this stand, but also ensured that study trees were outside the influence of neighbouring trees and well outside the influence of nearby experimental plots, while still being on the same soil type. While longleaf pine are known to have extensive lateral root systems that extend well beyond the tree dripline (Hodgkins and Nichols 1977), the 30 m minimum spacing ensured hydraulic independence among these trees. Understory density was fairly uniform throughout the sampling area. We established two 4 m × 4 m plots ∼2.5 m from the base of each tree (Fig. 1). Although we considered setting up control plots on separate trees, we decided to establish control and treatment plots as pairs on the same tree to control as much as possible for effects of tree size, rooting depth, tree vigour, and soil texture on hydraulic lift, which could affect the outcome of the experiment. Plots were arranged in a blocked design, with each tree as a block and two plots (one trenched root exclusion plot and one untrenched control plot). The sparse canopy allows for >50% of daily ambient light to reach the understory; however, to control for the possibility of brief shading from direct sunlight, primarily by the tree bole, plots were randomly assigned to either the eastern or western side of each tree.

We conducted understory plant surveys in early May 2021 to identify the most abundant PFTs across the site. We selected study species (one from each of the four most abundant PFTs) based on commonness (presence in all plots) and relative abundance. Species selected were subshrubs of sand post oak (*Q. margaretta*, woody subshrub), wiregrass (*A. beyrichiana*, C_4_ grass), silkgrass (*Pityopsis aspera*, forb), and butterfly pea (*Clitoria mariana*, legume). Henceforth, to avoid confusion with common names, we refer to each species by functional type (i.e. the oak subshrub, the legume, and the forb) except for wiregrass. We randomly selected three healthy study individuals from each PFT within each plot. We tagged each unique individual at the beginning of the study and sampled the same individuals throughout the study. The forb, the legume, and the oak are all clonal species. For the purposes of this study, individuals that were located ≥1 m apart were assumed to be hydraulically independent. One ramet (the ‘main ramet,’ hereafter) was tagged on each individual of these three PFTs.

After diversity surveys were completed in late May, we trenched root exclusion treatment plots with an automatic trencher (Ditch Witch, Perry, OK, USA) to a depth of ∼1 m to sever tree roots and isolate treatment plots from tree roots. Trenched plots were 6 m × 6 m in size to allow for a 1-m buffer zone between the trench and the 4 m × 4 m experimental plot inside it (Fig. 1). We lined trenches with two layers of 4 mm plastic sheeting with a layer of soil about 15 cm thickness between them, following the methods of Pecot et al. (2007). Based on site characteristics and a previous study (Belovitch et al. 2022), we assumed the effects of trenching alone on volumetric water content (VWC) to be minimal. Because the slope at the site is negligible (0%–1%) and soils are well drained, lateral flow into or out of plots is negligible, regardless of the presence of a plastic liner. Therefore, any differences in soil moisture between the treatments should be driven by differences in plant uptake/evapotranspiration (*E*_t_) and/or hydraulic lift, although we did not measure *E*_t_ or HL directly in this study.

### Environmental and physiological measurements

We monitored soil moisture from May 2021 to January 2022 using new Teros10 probes, which were factory calibrated for mineral soils with an accuracy of ±0.03 m^3^ m^−3^ (3% VWC) for the sandy soils we measured (METER Group, Pullman, WA, USA). We installed two probes horizontally in each of the eight plots at depths of 10 and 50 cm (Fig. 1) and logged data every half hour with Em50 loggers (METER Group). Rainfall was measured at the site every half hour by a tipping bucket rain gauge (Onset, Bourne, MA, USA).

We took physiological measurements biweekly from June through September and weekly from October through mid-November (i.e. late growing season). We began in June because plants were fully leafed-out, leaves were large enough to fill the chamber for photosynthetic measurements, and plants were large enough to sustain repeated leaf harvest for measuring Ψ. We measured predawn and midday Ψ on study PFTs using a Schölander-type pressure chamber (PMS Instrument Co., Corvallis, OR, USA). When possible, we measured predawn Ψ on two to three leaves of the same ramets used for gas exchange but had to use other ramets for all midday measurements and for late-season (after October 1) predawn measurements to prevent overharvesting of leaves.

We measured photosynthesis (net carbon assimilation, or *A*_net_), stomatal conductance to water vapour (*g*_s_), and water use efficiency (WUE, *A*_net_/E) with a Li-COR 6400XT Portable Photosynthesis System (Li-COR, Lincoln, NE, USA) on the same days we measured Ψ. Chamber CO_2_ concentration was set to 410 ppm to reflect ambient conditions. Photosynthetic photon flux density (PPFD) was set at 1000 µmol m^2^ s^−1^ for the C_3_ PFTs (the forb, the legume, and the oak) and 2000µmol m^2^ s^−1^ for wiregrass. These settings ensured light saturation for each photosynthetic system represented, i.e. C_3_ for the forb, legume, and oak and C_4_ for the wiregrass (Brantley et al. 2024). We maintained relative humidity in the chamber between 50% and 80%, and temperatures were kept as close to ambient as possible. Since wiregrass leaves are very thin, we filled the chamber with one layer of leaves. Leaves were arranged side by side in a single layer to minimize gaps and overlaps. For some forb individuals, leaves were not large enough to fill the chamber; in these instances, we measured one forb basal leaf at a time, for which we then obtained the area to calculate physiological measurements on a per-area basis. During the first measurement period, we measured all three individuals of each PFT in each plot. On subsequent measurement periods, we had to reduce the number of individuals measured to two per PFT due to time constraints. Measurements occurred between 9 a.m. and 2 p.m. over the course of two consecutive days with similar weather conditions. During the final measurement period in mid-November, we took measurements on the newest sun leaves at the top of the plant on the oaks because the older leaves were senescing.

### Growth measurements

To assess changes in plant growth over the course of the experiment, we measured individual plants in the early, middle, and late growing season (June, August, and November) and compared relative growth over the course of the study to account for differences in plant size at the beginning of the study. Due to wide variations in growth forms among the study PFTs, we used different growth metrics for each. For wiregrass, we measured the circumference at the base of each tussock. For the oak and the forb, we measured the height of the main ramet up to the terminal bud. For the legume, we measured the length of the main ramet to the terminal node. We also measured the basal diameter of each oak main ramet by averaging two diameter measurements taken 90° from one another at the base of the stem, just above the basal swelling.

For the forb, we also counted the total number of ramets, leaves per main ramet, and total number of leaves on each individual ramet during both the initial and final measurement periods. Some of the flowering stalks had senesced by the final measurement period, but there were basal leaves present for all individuals. Similarly, most of the aboveground legume biomass had senesced by the final measurement period. At the end of the growing season (mid-November), we harvested aboveground tissue of wiregrass, oak, and forb (there was not enough legume material to collect by this time). For the wiregrass, we clipped the leaves at the base of the crown (current season’s growth). For the forb and the oak, we clipped the entire shoot as close to the ground as possible. Because the forb and the oak are both clonal, we also harvested secondary ramets originating from the same genet as the main ramet. Due to the patchier nature of the forb growth, it was more difficult to identify the genets. For this PFT, we placed a 0.1-m^2^ quadrat around the main ramet, with the main ramet directly in the centre of the square, and harvested all aboveground the forb biomass that fell within the square. We weighed the main ramets and secondary ramets for each individual plant separately, but we combined all aboveground biomass sampled from each individual when calculating final biomass. We dried all individuals in a drying oven at 70°C for 72 h before weighing.

### Statistical analyses

We conducted all analyses in R (version 3.6.3), in the RStudio environment. We assessed differences in soil moisture between treatments using a linear mixed-effects model (function ‘lme’ in the ‘nlme’ package, Pinheiro et al. 2020) with treatment as the fixed effect and block as a random intercept with an autocorrelation structure of order one. We first detrended the data from the effects of precipitation by subtracting the daily mean volumetric water content for all treatments and blocks (grand mean) from individual plot means, then finding the weekly average of these daily differences. We analysed the two probe depths separately.

We analysed physiological responses using a repeated measures approach, with individual plants as replicates where appropriate. We used linear mixed-effects models for these data as well (‘lmer’ function in package ‘lme4’) (Bates et al. 2015). We tested the fixed effect of treatment on Ψ, *A*_net_, *g*_s_, and WUE values for each PFT, with block and plant individual specified as random intercepts. To remove the seasonal signal from the data, we detrended all time-series by finding the grand mean (mean daily value for each PFT) and subtracting the PFT-specific value from each plant individual’s daily value. We did not include plant individual as a term in the midday Ψ models since different individuals were sampled each time. We also tested for the effect of soil moisture at different depths on *A*_net_, *g*_s_, and WUE data using the same model framework, and the volumetric water content reading closest in time to each gas exchange measurement.

Finally, we tested the effect of treatment on biomass and the effect of treatment and sampling date on the other growth metrics to determine whether PFTs increased in size over the course of the growing season. We conducted *post hoc* comparisons of means using the ‘emmeans ()’ function in the ‘emmeans’ package (Lenth 2021), correcting for multiple comparisons with the Tukey method. For all models, we removed random effects if they explained no (or negligible) variance and used Satterthwaite’s degrees of freedom method in the ‘lmerTest’ package (Kuznetsova et al. 2017). If necessary, we transformed data with either the natural log or square root function to meet test assumptions of normally distributed residuals.

## Results

### Treatment effects

**Figure 2:**
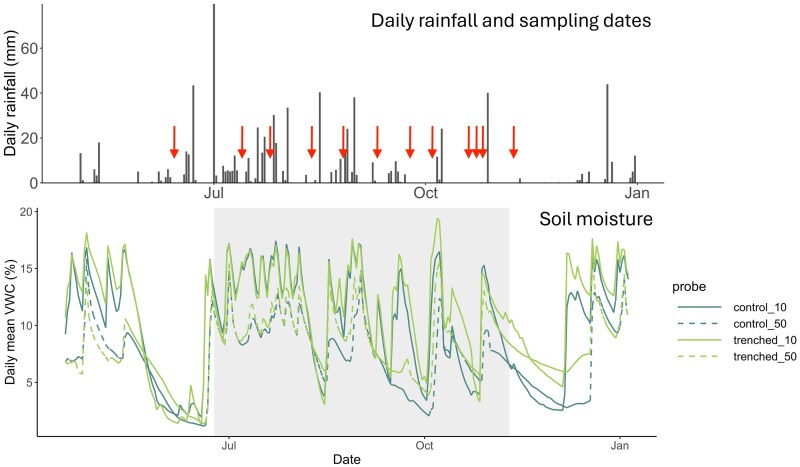
Total daily rainfall in mm over the course of the 2021 growing season (top panel) and daily volumetric water content at 10 and 50 cm depth in trenched root exclusion plots and untrenched control plots, averaged across all blocks (bottom panel). Red arrows in the top panel indicate the timing of gas exchange measurements. The grey shaded area in the bottom panel shows the same study period overlaying the soil moisture measurements.

Cumulative rainfall for the study period was 565 mm (Fig. 2; Supplementary Fig. S1), which was 53% higher than normal for the months of July–November, based on a 20-year mean (2001–2020) on Ichauway. Soil moisture at 10 cm depth did not differ between trenched root exclusion and untrenched control plots (*t*_250_ = 1.53, *P* = 0.13) (Fig. 2). Trenched plots were significantly wetter than control plots at 50 cm depth (*t*_247_ = 4.44, *P* < 0.001), with the overall mean difference of 1.1% VWC (9.5% in the control plots and 10.6% in the trenched plots). However, this difference was within the range of accuracy for probes we used and may not represent a definitive difference in soil moisture due to the treatments.

### Physiological measurements

**Figure 3:**
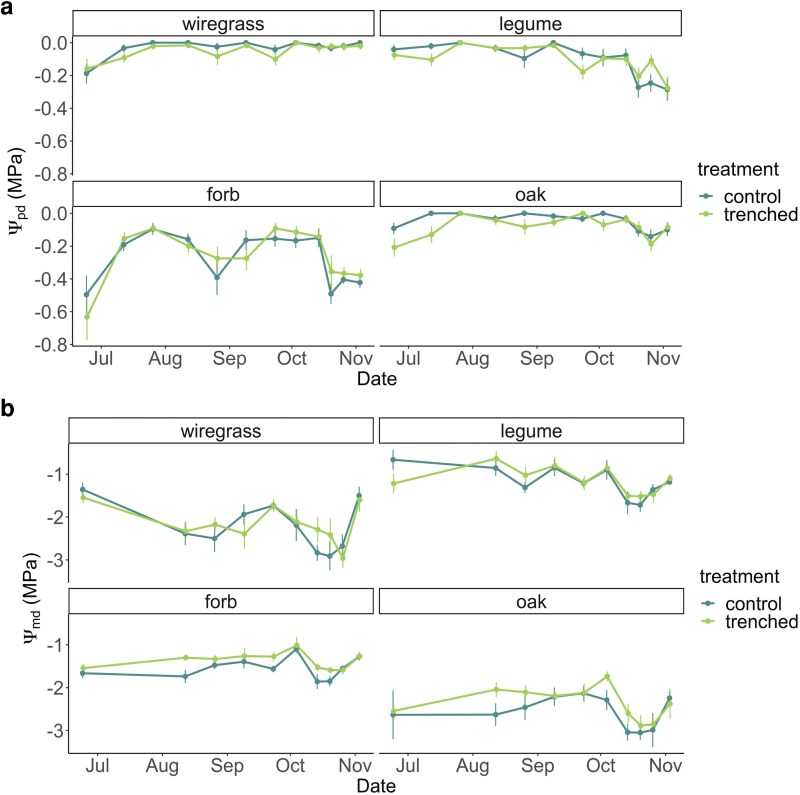
Predawn (a) and midday (b) leaf water potentials (mean ± SE) for each PFT over the course of the growing season. Plants in trenched root exclusion plots are represented by light green points, while plants in untrenched control plots are in dark green.

Predawn Ψ did not differ between treatments for any of the PFTs except for oak (*F*_1,275_ = 9.70, *P* = 0.002) for which Ψ were just 0.04 (±0.01) MPa higher in trenched plots than in control plots (Fig. 3a). Predawn Ψ remained high (∼ −0.2 MPa) over most of the growing season for all PFTs except for the forb (Fig. 3a). The forb experienced the lowest predawn Ψ (∼ −0.7 MPa) at the beginning of the growing season, but Ψ remained high for the forb thereafter, until a small dip after mid-October. No other PFT experienced predawn Ψ below −0.4 MPa at any point during the study. Midday Ψ differed by treatment for the oak (*t*_129.20_ = 2.28, *P* = 0.02) and the forb (*t*_140.08_ = 3.99, *P* < 0.001), but not for wiregrass (*t*_141_ = 0.31, *P* = 0.76) or the legume (*t*_141.05_ = 0.43, *P* = 0.67) (Fig. 3b). Midday Ψ values were 0.10 (±0.04) MPa higher in the trenched plots than controls for oak and 0.18 (±0.05) MPa higher for the forb.

**Figure 4:**
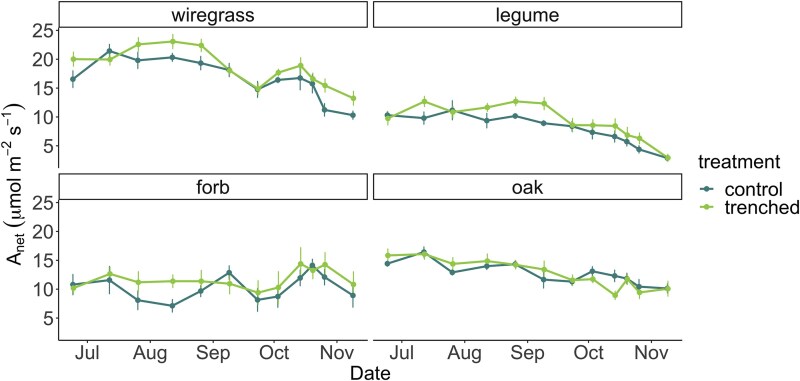
Photosynthesis (µmol m^2^ s^−1^; mean ± SE) at each sampling period for the four PFTs. Light green points represent plants in trenched root exclusion plots, while darker green data represent plants in untrenched control plots.

**Figure 5:**
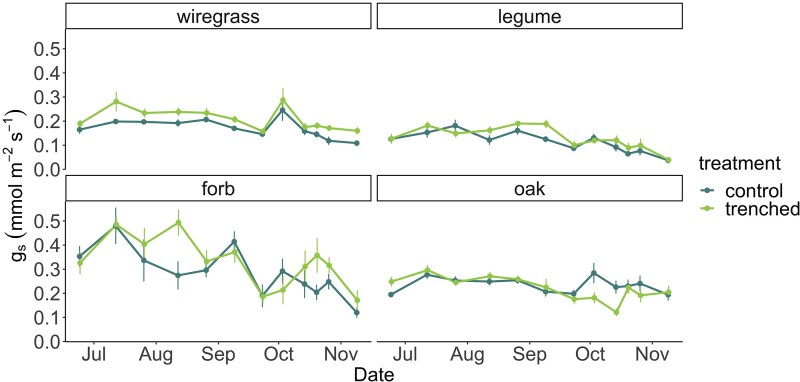
Stomatal conductance (mmol H_2_O m^2^ s^−1^; mean ± SE) at each sampling period for the four PFTs. Light green points represent plants in trenched root exclusion plots, while darker green data represent plants in untrenched control plots.

**Figure 6:**
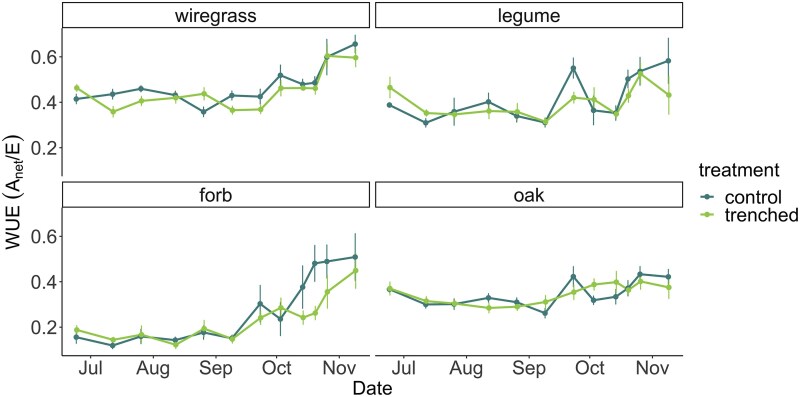
Water use efficiency (µmol C/mmol H_2_O) measurements (mean ± SE) at each sampling period for the four PFTs. Light green points represent plants in trenched root exclusion plots, while darker green data represent plants in untrenched control plots.

Comparing gas exchange, *A*_net_ differed significantly by treatment for wiregrass (*t*_22.81_ = 2.35, *P* = 0.03) and the legume (*t*_19.30_ = 2.589, *P* = 0.02), but not for the oak (*t*_23.73_ = 0.184, *P* = 0.86) or the forb (*t*_19.18_ = 0.59, *P* = 0.56) (Fig. 4). When averaged over the entire growing season, *A*_net_ was 1.6 μmol m^2^ s^−1^ higher in trenched plots for wiregrass and 1.3 μmol m^2^ s^−1^ higher for the legume. Similarly, *g*_s_ differed significantly by treatment for wiregrass (*t*_22.84_ = 3.71, *P* = 0.001) and the forb (*t*_18.99_ = 2.12, *P* = 0.055), but not for the legume (*t*_22.55_ = 1.65, *P* = 0.11) or the oak (*t*_19.82_ = 0.58, *P* = 0.57) (Fig. 5). When averaged over the entire growing season, *g*_s_ was 37 μmol m^2^ s^−1^ higher in trenched plots for wiregrass and 43 μmol m^2^ s^−1^ higher for the forb. There was no significant difference in WUE between treatments for any of the PFTs, although there was substantial seasonal variation with WUE increasing later in the growing season for all PFTs (Fig. 6).

### Soil moisture and gas exchange

All gas exchange parameters were significantly related to soil moisture at 50 cm depth, and the majority were related to soil moisture at 10 cm depth (Table 1). Both *A*_net_ and *g*_s_ increased with higher soil moisture while WUE declined with higher soil moisture (Table 1). *A*_net_ was positively related to VWC at 10 cm for wiregrass (*t*_164.67_ = 3.21, *P* = 0.02), oak (*t*_172.83_ = 2.31, *P* = 0.022), and the legume (*t*_156.89_ = 5.81, *P* < 0.001), but not for the forb (*t*_165.92_ = 0.37, *P* = 0.71) (Table 1). *A*_net_ was also positively related to VWC at 50 cm for all PFTs [wiregrass: (*t*_174.45_ = 4.34, *P* < 0.001), oak: (*t*_178.71_ = 3.99, *P* < 0.001), forb: (*t*_169.99_ = 2.19, *P* = 0.03), legume: (*t*_167.42_ = 3.87, *P* < 0.001); Table 1]. Stomatal conductance (*g*_s_) was positively related to VWC at 10 cm for the forb (*t*_161.65_ = 2.59, *P* = 0.008) and the legume (*t*_162.16_ = 5.88, *P* < 0.001), but not for wiregrass (*t*_160.64_ = 1.53, *P* = 0.13) or oak (*t*_159.38_ = 0.06, *P* = 0.95) (Table 1). Stomatal conductance was positively related to VWC at 50 cm for all PFTs [wiregrass: (*t*_170.71_ = 2.07, *P* = 0.04), oak: (*t*_177.54_ = 2.06, *P* = 0.041), forb: (*t*_161.12_ = 3.99, *P* < 0.001), legume: (*t*_166.07_ = 3.30, *P* = 0.001); Table 1]. WUE was negatively related to soil moisture for all plants at both 10 and 50 cm (all *P* < 0.05; Table 1).

**Table 1:** Relationship sign (+ or −) and marginal *r*^2^ values for relationships between photosynthesis (*A*_net_), stomatal conductance (*g*_s_), or WUE and soil volumetric water content in the top 10 or top 50 cm soil for each PFT.^a^

Physiological parameters for each functional type	10 cm	50 cm
*A*_net_		
Grass	+, 0.054*	+, 0.095*
Forb	0.001	+, 0.026*
Legume	+, 0.159*	+, 0.081*
Oak subshrub	+, 0.029*	+, 0.082*
*g*_s_		
Grass	0.012	+, 0.022*
Forb	+, 0.042*	+, 0.087*
Legume	+, 0.163*	+, 0.061*
Oak subshrub	<0.001	+, 0.023*
WUE		
Grass	−, 0.182*	−, 0.026*
Forb	−, 0.092*	−, 0.065*
Legume	−, 0.112*	−, 0.043*
Oak subshrub	−, 0.056*	−, 0.046*

^a^The slopes of the relationships were considered significantly different from zero at *P* < 0.05 (see text for more details on statistics) and are noted with an asterisk.

### Growth and biomass

There was no treatment effect on plant growth metrics (Table 2), and there was no significant difference in final biomass (total plant dry mass harvested at the end of the growing season) between trenched and control plots for any of the PFTs measured [wiregrass: (*t*_17.06_ = 1.39, *P* = 0.18); oak: (*t*_19_ = 0.94, *P* = 0.36); forb: (*t*_19_ = 0.66, *P* = 0.52); Table 2]. Wiregrass tussock circumference decreased over the course of the growing season for both treatment and control plots, but changes did not differ significantly between treatments [(*t*_24.05_ = 1.01, *P* = 0.32); Table 2]. Oak stem diameters increased in size throughout the study, but there was no effect of treatment on ramet diameter over the course of the season (*t*_34.95_ = 0.24, *P* = 0.81). There was also no effect of treatment on oak ramet height (*t*_34.38_ = 0.11, *P* = 0.913). Forb and legume height also increased over the course of the season in both trenched and control plots, but there was no significant difference in growth between the two treatments for either PFT (all *P* > 0.05; Table 2).

**Table 2:** Mean (±1 standard error) growth parameters for understory PFTs during one growing season.^a^

Growth parameter	Untrenched control	Trenched/root exclusion	Significant treatment effect?
End of season biomass (g)			
Wiregrass	81.19 ± 23.28	112.5 ± 26.01	No
Oak subshrub	47.21 ± 11.22	70.47 ± 25.75	No
Forb	5.52 ± 1.60	6.73 ± 1.07	No
Other growth parameters			
Change in wiregrass tussock circumference (mm)	−42.0 ± 23.7	−4.72 ± 29.80	No
Oak subshrub stem change in diameter (mm)	3.90 ± 1.23	4.11 ± 1.01	No
Oak subshrub change in height (cm)	24.94 ± 6.21	31.08 ± 8.34	No
Legume change in height (cm)	7.81 ± 5.83	5.87 ± 1.73	No
Forb change in height (cm)	25.13 ± 3.87	25.69 ± 3.15	No

^a^Differences in plant growth between trenched root exclusion plots and untrenched control plots were considered significant at *P* < 0.05 (see text for more details on statistics).

## Discussion

We predicted that plants in untrenched plots would demonstrate less water stress because of higher soil moisture, have higher stomatal conductance and net photosynthesis, and have higher growth rates due to the potential facilitative effects of mature longleaf pine trees on groundcover PFTs. These hypotheses, however, were not supported. Our data for soil moisture, understory plant physiology, and growth showed either no effect or only a slight increase in soil moisture and gas exchange across PFTs in trenched root exclusion plots. Although our study plants did not experience severe water stress during our study period, gas exchange was sensitive to soil moisture at 50 cm for all PFTs. Both *A*_net_ and *g*_s_ were positively related to volumetric water content, and WUE increased with decreasing soil moisture for all PFTs. These results suggest that the plants in our study were responding to differences in soil moisture through time and that water limitation is important for understory plants in this system. However, unlike previous studies at the same site, our data showed that root exclusion from trenching had a neutral or slightly positive effect on plant physiology by reducing competition, rather than a negative effect from excluding facilitative effects (Belovitch et al. 2024).

These results should be interpreted with some caution due to the low number of plots, limited geographic area of the study, and limited length of the study. The effort to trench these plots, find plots that had similar species assemblages, and regularly monitor physiology and growth of four species in just these four plots was considerable. However, these results are consistent with the findings of other studies in finding competitive interactions (Pecot et al. 2007, Barron-Gafford et al. 2021). They specifically agree with Barron-Gafford et al. (2021) who found that leaf-level gas exchange values in grasses and plot-level ecosystem productivity were greater in trenched plots during a year with high rainfall. By severing tree roots during trenching, we isolated plants from competition with longleaf pine for soil resources, and this outweighed any potential benefit from facilitative effects, such as subsidies from hydraulic lift.

### Effects of root exclusion on PFTs

Overall, predawn water potentials in our study were higher (i.e. less negative) than those observed for a similar suite of PFTs in Belovitch et al. (2024). The oak and forb showed some treatment effects on predawn Ψ, but the differences were small and inconsistent across time. We saw the same pattern among PFTs for midday Ψ with the oak and forb appearing more sensitive to treatment effects. But again, the direction of these effects was in the opposite direction to what we predicted and indicated a negative effect of root competition in untrenched plots. By mid-October, most PFTs experienced a noticeable decline in midday Ψ. This pattern was the same for both treatments and was likely more related to leaf age and phenology than soil moisture or treatment (Giencke et al. 2018).

Gas exchange also varied among PFTs with overall higher gas exchange in trenched plots for wiregrass, mixed effects for the legume and forb, and no effect for oaks. Differences among PFTs between the effects of trenching on Ψ and gas exchange suggest that water stress may not be the primary driver of gas exchange during this study. For example, because wiregrass showed no benefit from trenching in terms of Ψ but did show a significant benefit in terms of higher gas exchange rate, we suggest that this could indicate a potential nutrient limitation for grasses in control plots (Evans and Seemann 1989, Shen et al. 2024), but we did not measure leaf nutrient content across treatments for any functional group. The mixed results in the forb and legume (i.e. one showing higher *A*_net_ and the other higher *g*_s_ in trenched plot) suggest a slight impact of water stress in the legume and a slight effect of nutrient competition in the forb, but these results are not definitive. Finally, we suggest that the oak showed no treatment effect on gas exchange. This could be because these individuals likely have a high root:shoot ratio, which might mitigate effects of root competition in control plots (Poorter et al. 2012). These oaks, although appearing as seedlings aboveground, have been repeatedly ‘top-killed’ by prescribed fire and have substantial roots systems relative to their aboveground biomass and leaf area (V. Hudspeth, unpublished data).

The marginally higher physiological performance of some PFTs in trenched plots did not lead to higher plant growth rates in the trenched plots for any PFT. By contrast, Pecot et al. (2007) reported increased hardwood seedling growth in trenched plots at a nearby site and attributed this to the removal of competition with adult pine trees for belowground resources. Other studies have also shown that excluding longleaf pine roots from gaps increases nutrient availability and root mass production of non-pine species (Jones et al. 2003). Because the differences we observed in gas exchange between trenched and reference plots were not large enough to result in measurable differences in aboveground growth, the effect of competition must have been minimal in our study, likely due to the unseasonably wet growing season.

### Rainfall effects on competition and facilitation

Higher than normal precipitation can reduce facilitative benefits on understory plants by triggering a shift towards tree-understory competition (Barron-Gafford et al. 2017). Longleaf pines, like many trees, can alter patterns of root water uptake based on growing conditions (Dawson and Pate 1996, Scholz et al. 2008, Belovitch et al. 2022). During drier periods, these trees may rely on deeper soil water, while during wetter periods, they may favour water uptake in shallow soils (Belovitch et al. 2022). With higher-than-normal rainfall during this study leading to higher water content in the shallow soil layers, the extensive lateral roots of the pines may have been more effective at taking up shallow soil water than the understory PFTs, thus negatively affecting plant physiology in control plots. However, another trenching experiment during a drought year in longleaf pine stands with more mesic soils reported that trenched plots were wetter than control plots in the first 30 cm of soil (Pecot et al. 2007). They attributed this to the exclusion of pine roots from the trenched plots, contrasting the findings of Belovitch et al. (2024).

Although we did not measure HL in this study, higher rainfall during the study period may have prevented HL from occurring or from having a measurable positive effect on soil moisture, plant physiology, or plant growth. Soil moisture availability was likely higher during our study than during the Belovitch et al. (2022, 2024) studies at the same site. Direct comparison of soil VWC between studies was not possible due to the use of different sensors and sampling depths. However, in the current study, predawn Ψ for the wiregrass, oak, and legume were less negative than predawn values observed at the same site in Belovitch et al. (2024). Overall, our minimum observation of predawn Ψ in the oak, grass, and legume was > −0.3 MPa, suggesting that our study plants did not experience severe or prolonged water stress at any point during the growing season. This further suggests that the shallow soils at the study site, regardless of treatment, may not have been dry enough to induce HL, negating one of the primary facilitative effects of longleaf pine roots.

Several works have also suggested that variability in precipitation drives temporal changes in HL (Espeleta et al. 2004, Warren et al. 2007, Lee et al. 2018, Barron-Gafford et al. 2021). For example, Barron-Gafford et al. (2021) used trenching to show that understory photosynthesis rates and gross productivity were higher in control plots during dry years, when HL was the dominant type of hydraulic redistribution. But in a wet year, hydraulic descent was the dominant form of hydraulic redistribution and understory in trenched plots were more productive and had higher rates of photosynthesis. Lee et al. (2018) modelled HL by mesquite trees over 1 year and showed that HL occurred during the dry season and likely supplemented understory growth. But HL shifted to hydraulic descent (HD) during the monsoon season and led to a net competitive use of water over the course of the year. These temporal associations of facilitative provisioning of water (HL) with drier conditions and of competitive use or storage of water with wetter conditions over time echo a similar continental-scale spatial pattern described by Dohn et al. (2013), who found that net interactions between trees and grasses tended to be facilitative in drier regions and competitive in wetter regions.

### Summary and implications

Our study adds valuable information to a limited body of work on tree–groundcover interactions in the longleaf pine ecosystem. Although our study was limited in duration and scope, it supports conclusions from other studies across different longleaf sites that have demonstrated that trees can have either facilitative or competitive effects on groundcover through a variety of mechanisms (Pecot et al. 2007, Loudermilk et al. 2016, Belovitch et al. 2024). While Belovitch et al. (2024) demonstrated that HLW is used by groundcover species and can increase Ψ, Pecot et al. (2007) showed evidence of pine-understory competition in a study of trenched plots in a mesic longleaf pine woodland during a severe drought and suggested that the effect was due to depletion of deep-soil water reserves (Pecot et al. 2007). The contrasting outcomes among these studies suggest that precipitation, soil, or a combination of the two are important drivers of interspecific interactions in longleaf and other woodland ecosystems. Our study also showed that these differences can affect various PFTs to different degrees and offers clues to differences in competition for water versus competition for nutrients.

More broadly, these findings have implications for understanding how future climate variability will impact overstory-understory interactions in longleaf pine and other woodland and savanna ecosystems. Southeastern USA has been experiencing less frequent but more intense rainfall events over the past century, with summer months becoming slightly drier, with longer periods of no rainfall (Kunkel et al. 2013, Powell and Keim 2015). It is unclear how precipitation patterns will change over the coming century. Some models predict that rainfall will stay the same or increase in certain parts of the region while other simulations show rainfall becoming more variable, with both very wet years and very dry years (Wuebbles et al. 2014, McNulty et al. 2019). For example, if climate change induces longer and/or more severe droughts, facilitative effects on understory plant survival and growth in these drier years might be a critical factor in maintaining groundcover diversity (Belovitch et al. 2024). However, these same effects might not occur at all during wetter years, and competition between canopy trees and understory species would dominate species interactions, affecting different PFTs to varying degrees and potentially reducing groundcover diversity in woodlands. Given the greatly diminished extent of longleaf ecosystems with intact groundcover communities in southeastern USA, understanding potential climate-induced shifts in canopy–groundcover interactions will be important for predicting how both remaining and restored stands respond to management and climate change.

## Supplementary Material

plag032_Supplementary_Data

## Data Availability

Data and code are available as Supporting Information.
