# Supplementary material for: Variable responses to tree root exclusion by understory plant functional types in a xeric longleaf pine woodland
Source: AoB Plants. 2026 Jul 18;18(4):plag032. doi: 10.1093/aobpla/plag032 (PMC13387064; doi:10.1093/aobpla/plag032)

Figure S1. Comparison of cumulative annual rainfall at a climate station ~5 km from the study site for 2021 (black line) and for all years 2001–2024 (gray lines). The red line shows the mean, and the standard deviation is shown in pink. The study period is shown by the gray box. Rainfall data are from the Georgia Automated Environmental Monitoring Network (www.georgiaweather.net).

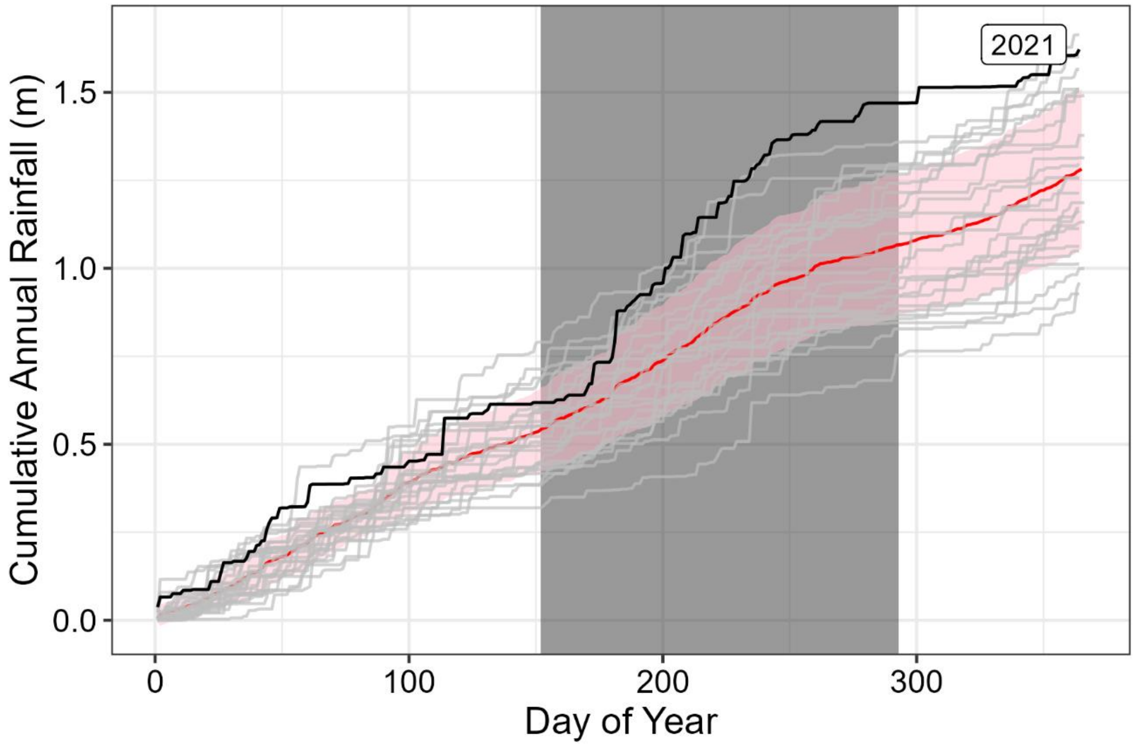

Supplement: plag032_Supplementary_Data [file plag032_supplementary_data.zip › Figure S1.pdf]
