# Supplementary material for: Variable responses to tree root exclusion by understory plant functional types in a xeric longleaf pine woodland
Source: AoB Plants. 2026 Jul 18;18(4):plag032. doi: 10.1093/aobpla/plag032 (PMC13387064; doi:10.1093/aobpla/plag032)
Supplement: plag032_Supplementary_Data [file plag032_supplementary_data.zip › Judge_analyses_code_2026.pdf]

**Soil moisture data manipulation & joining with gas exchange data**

```
# SM data analysis part 2: combining SM and gas ex data
# as of 9/3/21 (T10 data starts at 5/28)

library(tidyr)
library(dplyr)
library(reshape2)
library(stringr)
library(nlme)
library(timetk)
library(lubridate)
library(ggplot2)
#library(data.table) # v1.9.6+
library(padr)
library(MuMIn)


# combining data
T1_data <- read.csv("~/Documents/UGA /Grad Project/soil moisture:rainfall
data/compiled data/CSVs/T1_1.9.22_compiled_CSV.csv")
T4_data <- read.csv("~/Documents/UGA /Grad Project/soil moisture:rainfall
data/compiled data/CSVs/T4_1.9.22_compiled_CSV.csv")
T7_data <- read.csv("~/Documents/UGA /Grad Project/soil moisture:rainfall
data/compiled data/CSVs/T7_11.18.compiled_TRUNCATED.csv")
T10_data <- read.csv("~/Documents/UGA /Grad Project/soil moisture:rainfall
data/compiled data/CSVs/T10_1.9.22_cleaner.csv")

soil.moist <- rbind(T1_data,T4_data,T7_data,T10_data)

# put all data into long format
soil.moist.long <- melt(soil.moist, measure.vars = 4:7)
soil.moist.long <- rename(soil.moist.long, probe = variable)
soil.moist.long <- rename(soil.moist.long, vwc = value)
#head(soil.moist.long)

# adding treatment column
soil.moist.long$treatment = str_sub(soil.moist.long$probe,1,6)

# deleting 999s (NAs)
soil.moist.clean <-soil.moist.long[!soil.moist.long$vwc==999,]

# changing date (if need be)
soil.moist.clean$Date <- ymd(soil.moist.clean$Date)
```

```
# - - - - - - - - - - - - - - - - - - - - - - - - - - - - - - - - - - -
# Gas Ex dataset

# filtering out anything Tleaf>40deg, RHsample 50<x<80, Ci between 150 and 400, Ci/Ca
0<x<1, photo, cond >0.
# also creating new column for species
# also deleting unnecessary columns

gasex1 <- read.csv("~/Documents/UGA /Grad Project/gas exchange
data/cleaned_and_complete_gasex_1_6_22_csv.csv")
str(gasex1)

gasex_clean <- gasex1 %>%
  filter(Tleaf<40 & RH_S>50 & RH_S<80 & Ci>150 & Ci<400 & Ci.Ca<1 & Photo>0 & Cond>0)
%>%
  mutate(species = str_sub(plant_ind,1,4)) %>%

select(date,HHMMSS,treatment,tree,species,plant_ind,WUE,Photo,Cond,Trans,VpdL,Vpd
A,Tair,Tleaf,RH_R,RH_S,PARo) %>%
  as.data.frame()

library(lubridate)

gasex_clean.2 <- gasex_clean
gasex_clean.2$date <- mdy(gasex_clean.2$date)

gasex_clean.2$datetime <- paste(gasex_clean.2$date, gasex_clean.2$HHMMSS)
gasex_clean.2$datetime <- as_datetime(gasex_clean.2$datetime)

gasex_clean.2$hour <- as.integer(format(gasex_clean.2$datetime, '%H'))
gasex_clean_before2 <- subset(gasex_clean.2, hour <= 14)

gasex_clean$tree <- toupper(gasex_clean$tree)
gasex_clean.2$tree <- toupper(gasex_clean.2$tree)

# before 2pm!
gasex_clean.2$hour <- as.integer(format(gasex_clean.2$datetime, '%H'))
gasex_clean_before2<- subset(gasex_clean.2, hour <= 14)

# renaming spp
gasex_clean_before2$species <- as.factor(gasex_clean_before2$species)
levels(gasex_clean_before2$species) <- c("wiregrass", "legume", "forb", "oak")
```

```
# JOINING GAS EX AND SM DATA AT 30 MIN INTERVALS - - - - - - - - - - - - - - - - - - - - - - - - - - - -
- - - - - - - - - - -


# measurements closest in time DF
soil.moist.clean$Measurement_Time <-
as.character(soil.moist.clean$Measurement_Time)
#soil_moist_times <- soil.moist.clean %>% separate(Measurement_Time,
c("date2","time"), sep = " ")


soil.moist.clean$Measurement_Time <-mdy_hm(soil.moist.clean$Measurement_Time)
soil.moist.clean <- rename(soil.moist.clean, 'datetime' = 'Measurement_Time')


# subsetting
soil_moist_times <- soil.moist.clean %>% select(Tree, datetime, probe, vwc, treatment)

# putting gas ex times into 30min bins (goes to previous 30mins)
gasex_clean_times.2 <- gasex_clean_before2 %>%
  thicken(interval = "30 mins", by = "datetime")


# making treatment columns the same:
soil_moist_times$treatment[soil_moist_times$treatment=="trench"]<-"trenched"
soil_moist_times$treatment[soil_moist_times$treatment=="contro"]<-"control"

# joining gas ex and sm data (30 min bins)
gas_sm_joined <- left_join(gasex_clean_times.2, soil_moist_times, by =
c("datetime_30_min" = "datetime", "treatment" = "treatment", "tree" = "Tree"))

#adding a probe depth column
gas_sm_joined$probe <- as.character(gas_sm_joined$probe)

gas_sm_joined <- gas_sm_joined %>%
  mutate(probe_depth = case_when(endsWith(probe, "_10") ~ "10cm", endsWith(probe,
"_50") ~ "50cm"))

gas_sm_joined$unique_ID <- paste(gas_sm_joined$tree, gas_sm_joined$treatment,
gas_sm_joined$plant_ind)
```

```
# Re-ordering data
gas_sm_joined_ordered <- gas_sm_joined[order(gas_sm_joined$tree,
gas_sm_joined$treatment, gas_sm_joined$plant_ind, gas_sm_joined$date),]
head(gas_sm_joined_ordered)


# separating into 10cm and 50cm depth dfs
ten_cm_gas_sm <-
gas_sm_joined_ordered[which(gas_sm_joined_ordered$probe_depth=='10cm'),]
fifty_cm_gas_sm <-
gas_sm_joined_ordered[which(gas_sm_joined_ordered$probe_depth=='50cm'),]


# separating data by spp
ten_cm_gas_sm_ARST <- ten_cm_gas_sm[which(ten_cm_gas_sm$species=='wiregrass'),]
fifty_cm_gas_sm_ARST <-
fifty_cm_gas_sm[which(fifty_cm_gas_sm$species=='wiregrass'),]


ten_cm_gas_sm_QUMA <- ten_cm_gas_sm[which(ten_cm_gas_sm$species=='oak'),]
fifty_cm_gas_sm_QUMA <- fifty_cm_gas_sm[which(fifty_cm_gas_sm$species=='oak'),]


ten_cm_gas_sm_PIGR <- ten_cm_gas_sm[which(ten_cm_gas_sm$species=='forb'),]
fifty_cm_gas_sm_PIGR <- fifty_cm_gas_sm[which(fifty_cm_gas_sm$species=='forb'),]


ten_cm_gas_sm_CLMA <- ten_cm_gas_sm[which(ten_cm_gas_sm$species=='legume'),]
fifty_cm_gas_sm_CLMA <- fifty_cm_gas_sm[which(fifty_cm_gas_sm$species=='legume'),]
```

**Soil moisture analysis**

```
############### vwc_diff models -################ - - - - - - - - - - - - - - -

# STEPS
# 1. find grand mean (mean VWC for each day) for either 10cm or 50cm soil depth
# 2. join this df with df that contains daily mean VWC for each probe
# 3. subtract grand mean from each probe's daily mean
# 4. grouped days into weeks with floor_date()
# 5. grouped the data by (Tree, treatment, WEEK, probe) (to get weekly average of the
differences between daily avg and daily grand mean)
# 6. Ordered by 1. tree, 2. treatment, 3. week


#10cm - - - -

# finding grand mean for just 10cm data
soil.moist.clean$probe <- as.character(soil.moist.clean$probe)

grand_mean10cm <- soil.moist.clean %>%
  mutate(probe_depth = case_when(endsWith(probe, "_10") ~ "10cm", endsWith(probe,
"_50") ~ "50cm")) %>%
  filter(probe_depth=="10cm") %>%
  group_by(Date)  %>%
  summarize(grand_mean = mean(vwc)) %>%
  as.data.frame()

# find daily avg VWC per unique probe
class(soil.moist.clean$probe)
soil.moist.clean$probe <- as.character(soil.moist.clean$probe)

vwc_byprobe <- soil.moist.clean %>%
  group_by(Date, Tree, treatment, probe) %>%
  summarize(mean_vwc = mean(vwc))  %>%
  mutate(probe_depth = case_when(endsWith(probe, "_10") ~ "10cm", endsWith(probe,
"_50") ~ "50cm"))
vwc_byprobe

class(vwc_byprobe$treatment)
vwc_byprobe$treatment <- as.factor(vwc_byprobe$treatment)

# separating into 10 and 50cm depths
ten_cm_sm <- vwc_byprobe[which(vwc_byprobe$probe_depth=='10cm'),]
fifty_cm_sm <- vwc_byprobe[which(vwc_byprobe$probe_depth=='50cm'),]
```

```
# joining mean daily vwc for each probe with mean daily vwc dfs.
soil.moist.joined_10cm <- left_join(ten_cm_sm, grand_mean10cm, by = c("Date" = "Date"))

# Turning date into an integer
soil.moist.joined_10cm$Date.int <- as.integer(soil.moist.joined_10cm$Date)
soil.moist.joined_10cm$Date.3 <- soil.moist.joined_10cm$Date.int - 18731

# subtracting daily grand mean from daily probe means
soil.moist.joined_10cm <- soil.moist.joined_10cm %>%
  mutate(vwc_diff_10cm = mean_vwc - grand_mean) %>%
  as.data.frame


# 10cm model VWC diff by day, grouped by week
## subtract the DAILY grand mean from daily means before grouping by week

#making date a date
soil.moist.joined_10cm$Date <- as.Date(soil.moist.joined_10cm$Date)

# creating a 'week' column
soil.moist.joined_10cm$week <- floor_date(soil.moist.joined_10cm$Date, unit = "week")
head(soil.moist.joined_10cm)

# grouping by week
soil.moist.joined_10cm_2 <- soil.moist.joined_10cm %>%
  group_by(Tree, treatment, week, probe) %>%
  summarize(mean_vwc_diff10 = mean(vwc_diff_10cm)) %>% #the weekly average of the
differences between daily avg and daily grand mean
  as.data.frame()

# reordering data
soil.moist.joined_10cm_3 <-
soil.moist.joined_10cm_2[order(soil.moist.joined_10cm_2$Tree,
soil.moist.joined_10cm_2$treatment, soil.moist.joined_10cm_2$week),]
head(soil.moist.joined_10cm_3)

# 10cm model
vwcDiff_10cm.mod_weekly3<- lme(mean_vwc_diff10 ~ treatment, random = ~ 1 | Tree,
correlation = corAR1(), data = soil.moist.joined_10cm_3)
summary(vwcDiff_10cm.mod_weekly3)
Anova(vwcDiff_10cm.mod_weekly3)

# residuals
```

```
qqnorm(residuals(vwcDiff_10cm.mod_weekly3)) # pretty good
qqline(residuals(vwcDiff_10cm.mod_weekly3))


# 50cm ------------------

# grand mean for each date
grand_mean50cm <- soil.moist.clean %>%
  mutate(probe_depth = case_when(endsWith(probe, "_10") ~ "10cm", endsWith(probe,
"_50") ~ "50cm")) %>%
  filter(probe_depth=="50cm") %>%
  #filter(vwc_diff < 2 & vwc_diff > -2) %>%
  group_by(Date)  %>%
  summarize(grand_mean = mean(vwc)) %>%
  as.data.frame()

# joining mean daily vwc for each probe with mean daily vwc dfs.
soil.moist.joined_50cm <- left_join(fifty_cm_sm, grand_mean50cm, by = c("Date" =
"Date"))

# subtracting daily grand mean from daily probe means
soil.moist.joined_50cm <- soil.moist.joined_50cm %>%
  mutate(vwc_diff_50cm = mean_vwc - grand_mean) %>%
  as.data.frame

# making date a date
soil.moist.joined_50cm$Date <- as.Date(soil.moist.joined_50cm$Date)

# grouping by week
soil.moist.joined_50cm$week <- floor_date(soil.moist.joined_50cm$Date, unit = "week")
head(soil.moist.joined_50cm)

soil.moist.joined_50cm_2 <- soil.moist.joined_50cm %>%
  group_by(Tree, treatment, week, probe) %>%
  summarize(mean_vwc_diff50 = mean(vwc_diff_50cm)) %>% #the weekly average of the
differences between daily avg and daily grand mean
  as.data.frame()
head(soil.moist.joined_50cm_2)

# 50cm vwc_diff model
soil.moist.joined_50cm_3 <-
soil.moist.joined_50cm_2[order(soil.moist.joined_50cm_2$Tree,
soil.moist.joined_50cm_2$treatment, soil.moist.joined_50cm_2$week),]
head(soil.moist.joined_50cm_3)
```

```
vwcDiff_50cm.mod_weekly3<- lme(mean_vwc_diff50 ~ treatment, random = ~ 1 | Tree,
correlation = corAR1(), data = soil.moist.joined_50cm_3)
summary(vwcDiff_50cm.mod_weekly3)
Anova(vwcDiff_50cm.mod_weekly3)
emm_vwcDiff_50 <- emmeans(vwcDiff_50cm.mod_weekly3, pairwise~treatment,
adjust="Tukey")
emm_vwcDiff_50$contrasts %>%
  confint()

# residuals
hist(residuals(vwcDiff_50cm.mod_weekly3))
qqnorm(diff_resid50cm_weekly)
qqline(diff_resid50cm_weekly)
```

**Anet, Gs, Water Use Efficiency as a function of VWC Models**

```
# Photosynthesis ~ Volumetric Water Content -------------

# 10cm

# wiregrass
Photo_vwc_10cm_RM_ARST  <- lmer(Photo~vwc + (1|unique_ID), data =
ten_cm_gas_sm_ARST) # removed tree
summary(Photo_vwc_10cm_RM_ARST)

# oak
Photo_vwc_10cm_RM_QUMA <- lmer(Photo~vwc + (1|unique_ID), data =
ten_cm_gas_sm_QUMA) # removed tree
summary(Photo_vwc_10cm_RM_QUMA)

# pigr
Photo_vwc_10cm_RM_PIGR <- lmer(Photo~vwc + (1|tree), data = ten_cm_gas_sm_PIGR) #
removed unique_id because explained 0 variance
summary(Photo_vwc_10cm_RM_PIGR)

#clma
Photo_vwc_10cm_RM_CLMA<- lmer(Photo~vwc + (1|tree) + (1|unique_ID), data =
ten_cm_gas_sm_CLMA)
summary(Photo_vwc_10cm_RM_CLMA)

# 50cm

# wiregrass
Photo_vwc_50cm_RM_ARST  <- lmer(Photo~vwc + (1|unique_ID), data =
fifty_cm_gas_sm_ARST) # removed tree
summary(Photo_vwc_50cm_RM_ARST)

#oak
Photo_vwc_50cm_RM_QUMA  <- lmer(Photo~vwc + (1|unique_ID), data =
fifty_cm_gas_sm_QUMA) # removed tree
summary(Photo_vwc_50cm_RM_QUMA)

#pigr
Photo_vwc_50cm_RM_PIGR <- lmer(Photo~vwc + (1|tree), data = fifty_cm_gas_sm_PIGR) #
removed unique_id...0 var. explained
summary(Photo_vwc_50cm_RM_PIGR)
```

```
#clma
Photo_vwc_50cm_RM_CLMA<- lmer(Photo~vwc + (1|tree), data = fifty_cm_gas_sm_CLMA)
# unique_ID var. explained = essentially 0
summary(Photo_vwc_50cm_RM_CLMA)


#### Cond ~ VWC -------------------

# 10cm

#arst
summary(gs_vwc_10cm_RM_ARST <- lmer(log(Cond)~vwc + (1|unique_ID), data =
ten_cm_gas_sm_ARST))
# quma
summary(gs_vwc_10cm_RM_QUMA <- lmer(log(Cond)~vwc + (1|tree) + (1|unique_ID), data
= ten_cm_gas_sm_QUMA))
# pigr
summary(gs_vwc_10cm_RM_PIGR <- lmer(log(Cond)~vwc + (1|tree), data =
ten_cm_gas_sm_PIGR)) #removed unique_id because it explained no variance
#clma
summary(gs_vwc_10cm_RM_CLMA <- lmer(Cond~vwc + (1|tree), data =
ten_cm_gas_sm_CLMA))


# 50cm

#arst
summary(gs_vwc_50cm_RM_ARST <- lmer(log(Cond)~vwc + (1|unique_ID), data =
fifty_cm_gas_sm_ARST))
#quma
summary(gs_vwc_50cm_RM_QUMA <- lmer(log(Cond)~vwc + (1|unique_ID), data =
fifty_cm_gas_sm_QUMA))
#pigr
summary(gs_vwc_50cm_RM_PIGR <- lmer(log(Cond)~vwc + (1|tree), data =
fifty_cm_gas_sm_PIGR))
#clma
summary(gs_vwc_50cm_RM_CLMA <- lmer(Cond~vwc + (1|tree), data =
fifty_cm_gas_sm_CLMA))


# WUE by VWC (by species):

# 10cm
```

```
# ARST
summary(WUE_vwc_10cm_RM_ARST <- lmer(WUE~vwc + (1|tree), data =
ten_cm_gas_sm_ARST))

#CLMA
summary(WUE_vwc_10cm_RM_CLMA <- lmer(sqrt(WUE)~vwc + (1|tree) + (1|unique_ID),
data = ten_cm_gas_sm_CLMA)) # removed tree
# sqrt transformed

#QUMA
summary(WUE_vwc_10cm_RM_QUMA <- lm(log(WUE)~vwc, data =
ten_cm_gas_sm_QUMA)) #LM
# log trans

# PIGR
summary(WUE_vwc_10cm_RM_PIGR <- lm(log(WUE)~vwc, data = ten_cm_gas_sm_PIGR))


# 50cm

# ARST
summary(WUE_vwc_50cm_mod_ARST <- lme(WUE~vwc, random = ~ 1 | tree, correlation =
corAR1(), data =  fifty_cm_gas_sm_ARST, na.action = na.omit))

summary(WUE_vwc_50cm_RM_ARST <- lmer(WUE~vwc + (1|tree), data =
fifty_cm_gas_sm_ARST)) # removed unique_ID

# CLMA
summary(WUE_vwc_50cm_RM_CLMA <- lmer(log(WUE)~vwc + (1|tree) + (1|unique_ID),
data = fifty_cm_gas_sm_CLMA)) # removed tree

# QUMA
summary(WUE_vwc_50cm_RM_QUMA <- lmer(log(WUE)~vwc + (1|unique_ID), data  =
fifty_cm_gas_sm_QUMA)) # removed tree

# PIGR
summary(WUE_vwc_50cm_RM_PIGR <- lmer(log(WUE)~vwc + (1|tree), data =
fifty_cm_gas_sm_PIGR)) # removed unique_ID # removed tree
```

**Water Potential data manipulation & analysis**

```
library(dplyr)
library(ggplot2)
install.packages("patchwork")
library(patchwork)
install.packages("statmod")
library(statmod)
library(lubridate)
library(lme4)
library(lmerTest)
install.packages("r2symbols")
library(r2symbols)


# Pre-dawns --------------------------

predawn_wps  <- read.csv("~/Documents/UGA /Grad
Project/WPs/predawn_WPs_CSV.csv")
str(predawn_wps)

# make a column in which everything between 0 and 1.5 bar is 0:

predawn_wps$MPa.2 <- predawn_wps$WP_MPa

predawn_wps$MPa.2[predawn_wps$MPa.2 >= -.15] <- 0

head(predawn_wps)

# removing NAs
predawn_wps.2 <- na.omit(predawn_wps)

predawn_wps.2$date <- mdy(predawn_wps.2$date)

# creating bout column and re-ordering data
predawn_wps.2 <- predawn_wps.2 %>%
  mutate(bout = case_when(date == "2021-06-24" | date == "2021-06-25" ~ "2021-06-24",
date == "2021-07-12" | date == "2021-07-13" ~ "2021-07-12",date == "2021-07-26" | date ==
"2021-07-27"  ~ "2021-07-26" ,date == "2021-08-12" | date == "2021-08-13"  ~ "2021-08-
12",date == "2021-08-26" | date == "2021-08-27"  ~ "2021-08-26" ,date == "2021-09-09" |
date == "2021-09-10"  ~ "2021-09-09" , date == "2021-09-23" | date == "2021-09-24"   ~
"2021-09-23" ,date == "2021-10-03" | date == "2021-10-04"  ~ "2021-10-03" ,date == "2021-
10-14" | date == "2021-10-15"  ~ "2021-10-14", date == "2021-10-20" | date == "2021-10-21"
```

```
~ "2021-10-20" ,date == "2021-10-26" | date == "2021-10-27"  ~ "2021-10-26", date ==
"2021-11-03" | date == "2021-11-04"  ~ "2021-11-03" ) ) %>%
  as.data.frame()

predawn_wps.2$bout <- as.Date(predawn_wps.2$bout)


# MIDDAYS ---------------------

midday_WPs_CSV <- read.csv("~/Documents/UGA /Grad
Project/WPs/midday_WPs_CSV.csv")
midday_wps <- midday_WPs_CSV
midday_wps<- na.omit(midday_wps)

midday_wps_clean <- midday_wps %>% mutate(WP_MPa2 = gsub("<","", WP_MPa))

# fixing date
midday_wps_clean$date <- mdy(midday_wps_clean$date)

# creating bout column and re-ordering data
midday_wps_clean  <- midday_wps_clean  %>%
  mutate(bout = case_when(date == "2021-06-24" | date == "2021-06-25" ~ "2021-06-24",
date == "2021-08-12" | date == "2021-08-13"  ~ "2021-08-12", date == "2021-08-26"  ~
"2021-08-26", date == "2021-09-09" | date == "2021-09-10"  ~ "2021-09-09", date == "2021-
09-23" | date == "2021-09-24"   ~ "2021-09-23", date == "2021-10-04"  ~ "2021-10-04", date
== "2021-10-14"  ~ "2021-10-14", date == "2021-10-20" | date == "2021-10-21"  ~ "2021-10-
20" ,date == "2021-10-26" | date == "2021-10-27"  ~ "2021-10-26" ,date == "2021-11-03" |
date == "2021-11-04"  ~ "2021-11-03"  ) ) %>%
  as.data.frame()

midday_wps_clean$bout <- as.Date(midday_wps_clean$bout)

midday_wps_clean$WP_MPa2 <- as.numeric(midday_wps_clean$WP_MPa2)
class(midday_wps_clean$WP_MPa2)


##### ANALYSIS #######
# STEPS
# 1. DETREND: find grand mean (mean WP for each day) for each species
# 2. join this df with df that contains daily mean WP for each plant (this is just one value
because I only took one measurement per day)
# 3. subtract grand mean from each plant's daily value
# 6. Order by 1. tree, 2. treatment, 3. date
```

```
# 7. Model structure: lme(mean_vwc_diff ~ treatment, random = ~ 1 | Tree, correlation =
corAR1(), data = xxx)

# step 1: detrend
head(predawn_wps.2)

PD_grand_mean <- predawn_wps.2 %>%
  group_by(date, species)  %>%
  summarize(grand_mean = mean(MPa.2)) %>%
  as.data.frame()

# step 2: join dfs
PDs_joined <- left_join(predawn_wps.2, PD_grand_mean, by = c("date" = "date", "species"
= "species")) %>%
  mutate(PD_diff = MPa.2 - grand_mean) %>%
  as.data.frame()
head(PDs_joined)

# step 3: separate by species
predawns_joined_ARST<- PDs_joined[which(PDs_joined$species=='ARST'),]
predawns_joined_CLMA<- PDs_joined[which(PDs_joined$species=='CLMA'),]
predawns_joined_QUMA<- PDs_joined[which(PDs_joined$species=='QUMA'),]
predawns_joined_PIGR<- PDs_joined[which(PDs_joined$species=='PIGR'),]

# step 6: order
# reordering data:
PDs_joined_ARST_ord <-
predawns_joined_ARST[order(predawns_joined_ARST$unique_ID,
predawns_joined_ARST$date),]
PDs_joined_CLMA_ord <-
predawns_joined_CLMA[order(predawns_joined_CLMA$unique_ID,
predawns_joined_CLMA$date),]
PDs_joined_QUMA_ord <-
predawns_joined_QUMA[order(predawns_joined_QUMA$unique_ID,
predawns_joined_QUMA$date),]
PDs_joined_PIGR_ord <- predawns_joined_PIGR[order(predawns_joined_PIGR$unique_ID,
predawns_joined_PIGR$date),]


## Models --------------------

summary(PD_RM_ARST <- lmer(sqrt(1-PD_diff) ~ treatment + (1|tree), data =
PDs_joined_ARST_ord))
```

```
summary(PD_RM_CLMA <- lmer(PD_diff ~ treatment  + (1| unique_ID), data =
PDs_joined_CLMA_ord))

summary(PD_RM_QUMA <- lm(sqrt(1-PD_diff) ~ treatment,  data =
PDs_joined_QUMA_ord))

summary(PD_RM_PIGR <- lmer(PD_diff ~ treatment + (1|tree) , data =
PDs_joined_PIGR_ord))

# middays -------------------------------

# middays -----------------------------------------
levels(midday_wps_clean$species) <- c("wiregrass", "legume", "forb", "oak")

# step 1:
MD_grand_mean <- midday_wps_clean %>%
  group_by(date, species)  %>%
  summarize(grand_mean = mean(WP_MPa2)) %>%
  as.data.frame()

# step 2: join dfs
midday_wps.3 <- na.omit(midday_wps_clean)
MDs_joined <- left_join(midday_wps.3, MD_grand_mean, by = c("date" = "date", "species"
= "species")) %>%
  mutate(MD_diff = WP_MPa2 - grand_mean) %>%
  as.data.frame()
head(MDs_joined)

# step 2.5: add column of unique individuals
MDs_joined$unique_ID <-
paste(MDs_joined$tree,MDs_joined$treatment,MDs_joined$species)

# step 3: separate by species
MDs_joined_ARST<- MDs_joined[which(MDs_joined$species=='ARST'),]
MDs_joined_CLMA<- MDs_joined[which(MDs_joined$species=='CLMA'),]
MDs_joined_QUMA<- MDs_joined[which(MDs_joined$species=='QUMA'),]
MDs_joined_PIGR<- MDs_joined[which(MDs_joined$species=='PIGR'),]

# step 6: order
# reordering data:
MDs_joined_ARST_ord <- MDs_joined_ARST[order(MDs_joined_ARST$unique_ID,
MDs_joined_ARST$date),]
MDs_joined_CLMA_ord <- MDs_joined_CLMA[order(MDs_joined_CLMA$unique_ID,
MDs_joined_CLMA$date),]
```

```
MDs_joined_QUMA_ord <- MDs_joined_QUMA[order(MDs_joined_QUMA$unique_ID,
MDs_joined_QUMA$date),]
MDs_joined_PIGR_ord <- MDs_joined_PIGR[order(MDs_joined_PIGR$unique_ID,
MDs_joined_PIGR$date),]


## Models ----------------------------------------

# RM ARST
summary(lmer(MD_diff ~ treatment + (1|tree), data = MDs_joined_CLMA_ord) )

summary(MD_RM_QUMA <- lmer(sqrt(1 - MD_diff) ~ treatment + (1|tree), data =
MDs_joined_QUMA_ord) )

summary(mixed.mod_MDs_PIGR<- lme(MD_diff ~ treatment, random = ~ 1 | tree,
correlation = corAR1(), data = MDs_joined_PIGR_ord))

summary(MD_RM_PIGR <-lmer(MD_diff ~ treatment + (1|tree), data =
MDs_joined_PIGR_ord) )
```

**Anet, Gs, and WUE ~ treatment**

```
library(dplyr)
library(lme4)
library(lmerTest)
library(ggplot2)
library(car)
library(patchwork)
library(reshape2)
library(stringr)
library(lubridate)
library(performance)

gasex1 <- read.csv("~/Documents/UGA /Grad Project/gas exchange
data/cleaned_and_complete_gasex_1_6_22_csv.csv")

# filtering out anything Tleaf>40deg, RHsample, 50<x<80, Ci between 150 and 400, Ci/Ca
0<x<1, photo, cond >0.
# also creating new column for species
# also deleting unnecessary columns

gasex_clean <- gasex1 %>%
  filter(Tleaf<40 & RH_S>50 & RH_S<80 & Ci>150 & Ci<400 & Ci.Ca<1 & Photo>0 & Cond>0)
%>%
  mutate(species = str_sub(plant_ind,1,4)) %>%

select(date,HHMMSS,treatment,tree,species,plant_ind,WUE,Photo,Cond,Trans,VpdL,Vpd
A,Tair,Tleaf,RH_R,RH_S,PARo) %>%
  as.data.frame()

gasex_clean$tree <- toupper(gasex_clean$tree)

# fixing the date
gasex_clean$date <-mdy(gasex_clean$date)
class(gasex_clean$date)

# just data taken before 2pm
gasex_before2pm <- gasex_clean %>%
  filter(HHMMSS<14)

gasex_clean_before2 <- gasex_clean_before2 %>%
  mutate(bout = case_when(date == "2021-06-24" | date == "2021-06-25" ~ "2021-06-24",
date == "2021-07-12" | date == "2021-07-14" | date == "2021-07-15" ~ "2021-07-12", date
== "2021-07-26" | date == "2021-07-27"  ~ "2021-07-26", date == "2021-08-12" | date ==
```

```
"2021-08-13"  ~ "2021-08-12" ,date == "2021-08-26" | date == "2021-08-27"  ~ "2021-08-26"
,date == "2021-09-09"  ~ "2021-09-09" , date == "2021-09-23" | date == "2021-09-24" ~
"2021-09-23", date == "2021-10-03" | date == "2021-10-04"  ~ "2021-10-03", date == "2021-
10-14" | date == "2021-10-15"  ~ "2021-10-14", date == "2021-10-20" | date == "2021-10-21"
| date ==  "2021-10-22"  ~ "2021-10-20", date == "2021-10-26" | date == "2021-10-27"  ~
"2021-10-26", date == "2021-11-09" | date == "2021-11-10"  ~ "2021-11-09"  ) ) %>%
  as.data.frame()

gasex_clean_before2$bout <- as.Date(gasex_clean_before2$bout)

# step 1: detrend
gasex_grand_spp_mean <- gasex_clean_before2 %>%
  group_by(date, species)  %>%
  summarize(grand_mean_Photo = mean(Photo), grand_mean_gs = mean(Cond)) %>%
  as.data.frame()

# step 1.5: make a df for daily photosynthesis and gs for each plant
gasex_means_by_ind <- gasex_clean_before2 %>%
  select(date, tree, treatment, species, plant_ind, Photo, Cond)  %>%
  as.data.frame()

# step 2: join dfs
test_2 <- left_join(gasex_means_by_ind, gasex_grand_spp_mean, by = c("date" = "date",
"species" = "species")) %>%
  mutate(Photo_diff = Photo - grand_mean_Photo, gs_diff = Cond - grand_mean_gs) %>%
  as.data.frame()

# subset into species dfs
test_2_ARST<- test_2[which(test_2$species=='wiregrass'),]
test_2_CLMA<- test_2[which(test_2$species=='legume'),]
test_2_QUMA<- test_2[which(test_2$species=='oak'),]
test_2_PIGR<- test_2[which(test_2$species=='forb'),]

# step 3: re-order
test_2_ARST_ord <- test_2_ARST[order(test_2_ARST$tree, test_2_ARST$treatment,
test_2_ARST$plant_ind, test_2_ARST$date),]
test_2_CLMA_ord <- test_2_CLMA[order(test_2_CLMA$tree, test_2_CLMA$treatment,
test_2_CLMA$plant_ind, test_2_CLMA$date),]
test_2_QUMA_ord <- test_2_QUMA[order(test_2_QUMA$tree, test_2_QUMA$treatment,
test_2_QUMA$plant_ind, test_2_QUMA$date),]
test_2_PIGR_ord <- test_2_PIGR[order(test_2_PIGR$tree, test_2_PIGR$treatment,
test_2_PIGR$plant_ind, test_2_PIGR$date),]

# first need to make unique_ind column
```

```
test_2_ARST_ord$unique_ID <-
paste(test_2_ARST_ord$tree,test_2_ARST_ord$treatment,test_2_ARST_ord$plant_ind)
test_2_CLMA_ord$unique_ID <-
paste(test_2_CLMA_ord$tree,test_2_CLMA_ord$treatment,test_2_CLMA_ord$plant_ind)
test_2_QUMA_ord$unique_ID <-
paste(test_2_QUMA_ord$tree,test_2_QUMA_ord$treatment,test_2_QUMA_ord$plant_ind)
test_2_PIGR_ord$unique_ID <-
paste(test_2_PIGR_ord$tree,test_2_PIGR_ord$treatment,test_2_PIGR_ord$plant_ind)


## WUE data manipulation
# step 1: detrend
gasex_grand_spp_mean_WUE <- gasex_clean_before2 %>%
  group_by(date, species)  %>%
  summarize(grand_mean_WUE = mean(WUE))  %>%
  as.data.frame()

# step 1.5: make a df for WUE for each plant
gasex_means_by_ind_WUE <- gasex_clean_before2 %>%
  select(date, tree, treatment, species, plant_ind, WUE)  %>%
  as.data.frame()

# joining:
WUE_joined <- left_join(gasex_means_by_ind_WUE, gasex_grand_spp_mean_WUE, by =
c("date" = "date", "species" = "species")) %>%
  mutate(WUE_diff = WUE - grand_mean_WUE) %>%
  as.data.frame()

# making unique ID a column
WUE_joined$unique_ind <- paste(WUE_joined$tree,
WUE_joined$treatment,WUE_joined$plant_ind, sep="_")

# subset into species dfs
WUE_joined_ARST<- WUE_joined[which(WUE_joined$species=='wiregrass'),]
WUE_joined_CLMA<- WUE_joined[which(WUE_joined$species=='legume'),]
WUE_joined_QUMA<- WUE_joined[which(WUE_joined$species=='oak'),]
WUE_joined_PIGR<- WUE_joined[which(WUE_joined$species=='forb'),]


# step 3: re-order
WUE_joined_ARST_ord <- WUE_joined_ARST[order(WUE_joined_ARST$tree,
WUE_joined_ARST$treatment, WUE_joined_ARST$plant_ind, WUE_joined_ARST$date),]
WUE_joined_CLMA_ord <- WUE_joined_CLMA[order(WUE_joined_CLMA$tree,
WUE_joined_CLMA$treatment, WUE_joined_CLMA$plant_ind, WUE_joined_CLMA$date),]
```

```
WUE_joined_QUMA_ord <- WUE_joined_QUMA[order(WUE_joined_QUMA$tree,
WUE_joined_QUMA$treatment, WUE_joined_QUMA$plant_ind,
WUE_joined_QUMA$date),]
WUE_joined_PIGR_ord <- WUE_joined_PIGR[order(WUE_joined_PIGR$tree,
WUE_joined_PIGR$treatment, WUE_joined_PIGR$plant_ind, WUE_joined_PIGR$date),]


### Models -------------------------

# Anet ------------------
summary(RM_Photo_ARST <- lmer(Photo_diff ~ treatment + (1|unique_ID), data =
test_2_ARST_ord)) # removed tree, no influence

summary(RM_Photo_CLMA <- lmer(Photo_diff ~ treatment + (1|tree) + (1|unique_ID), data =
test_2_CLMA_ord))  # tree and unique id don't really have much of an effect

summary(RM_Photo_QUMA <- lmer(Photo_diff ~ treatment + (1|unique_ID), data =
test_2_QUMA_ord))

summary(RM_Photo_PIGR <- lmer(sqrt(abs(Photo_diff)) ~ treatment + (1|tree), data =
test_2_PIGR_ord)) # unique id not important


# Cond ---------------------

# RM models
summary(RM_Cond_ARST <- lmer(gs_diff ~ treatment + (1|unique_ID), data =
test_2_ARST_ord)) # removed tree, no influence

summary(RM_Cond_CLMA <- lmer(gs_diff ~ treatment + (1|unique_ID), data =
test_2_CLMA_ord))  # tree

summary(RM_Cond_QUMA <- lmer(gs_diff ~ treatment + (1|tree) + (1|unique_ID), data =
test_2_QUMA_ord))

summary(RM_Cond_PIGR <- lmer(gs_diff ~ treatment + (1|tree) + (1|unique_ID), data =
test_2_PIGR_ord)) # unique id not important


# WUE by species

summary(RM_WUE_ARST <- lmer(WUE_diff ~ treatment + (1|unique_ind), data =
WUE_joined_ARST_ord)) # removed tree, no influence
```

summary(RM_WUE_CLMA <- lmer(WUE_diff ~ treatment + (1|tree) + (1|unique_ind), data = WUE_joined_CLMA_ord)) # tree and unique id don't really have much of an effect

summary(RM_WUE_QUMA <- lmer(WUE_diff ~ treatment + (1|tree) + (1|unique_ind), data = WUE_joined_QUMA_ord))

summary(RM_WUE_PIGR <- lmer(WUE_diff ~ treatment + (1|tree) + (1|unique_ind), data = WUE_joined_PIGR_ord))

**Biomass data manipulation and analysis**

```
library(ggplot2)
library(ggpubr)
library(grid)
library(lme4)
library(lmerTest)
library(dplyr)
library(performance)
library(tidyr)
library(emmeans)
library(lubridate)
library(car)

# just total biomass for now:
prelim_ARST_final_biomass_data <- read.csv("~/Documents/UGA /Grad Project/biomass
data/final biomass data/prelim_ARST_final_biomass_data.csv")

# remove the 2 questionable ones
ARST_biomass <- subset(prelim_ARST_final_biomass_data, treatment!="control???" &
treatment!="trenched???")
#View(ARST_biomass)


##########  QUMAs ---------------------------------------------------
QUMA_biomass <- read.csv("~/Documents/UGA /Grad Project/biomass data/final
biomass data/prelim_QUMA_biomass_data.csv")

######### PIGRs -----------------------------------------------------
PIGR_biomass <- read.csv("~/Documents/UGA /Grad Project/biomass data/final biomass
data/prelim_PIGR_biomass_data.csv")


# Models: final biomass

#arst
summary(ARST_finbio_mod <- lmer(dry_wt_total~treatment + (1|tree), data =
ARST_biomass))

#quma
summary(QUMA_finbio_mod <- lmer(total_wt_g~treatment + (1|tree), data =
QUMA_biomass))

#pigr
```

```
summary(PIGR_finbio_mod <- lmer(dry_wt_total~treatment + (1|tree), data =
PIGR_biomass))


# ARST circumferences
ARST_circs <- read.csv("~/Documents/UGA /Grad Project/biomass
data/ARST_circumferences_CSV.csv")
ARST_circs$bout <- as.factor(ARST_circs$bout)

# arst circumferences
summary(arst_circ_mod <- lmer(circumference_cm ~ treatment*date + (1|unique_id), data
= ARST_circs))
emmeans(arst_circ_mod, pairwise~treatment*date, adjust= "Tukey")

# QUMA - - - - - - - - - - - - - - - - - - - - - - - - - - - - - - - - - - -

QUMA_growth<- read.csv("~/Documents/UGA /Grad Project/biomass
data/QUMA_growth_rate_csv.csv")
QUMA_growth$bout <- as.factor(QUMA_growth$bout)

# DBH Model
summary(quma_dbh_mod<- lmer(avg_ramet_dbh ~ treatment*bout + (1|tree) +
(1|unique_id), data = QUMA_growth))
quma_dbh_emm <- emmeans(quma_dbh_mod, pairwise~treatment*bout, adjust=
"Tukey")

# Height model
# quma ramet height
summary(quma_height_mod <- lmer(ramet_height ~ treatment*bout + (1|tree) +
(1|unique_id), data = QUMA_growth))
emmeans(quma_height_mod, pairwise~treatment*bout, adjust= "Tukey")


# CLMA - - - - - - - - - -
CLMA_heights <- read.csv("~/Documents/UGA /Grad Project/biomass
data/CLMA_heights_CSV.csv")

CLMA_heights$bout <- as.factor(CLMA_heights$bout)
CLMA_heights <- na.omit(CLMA_heights)
CLMA_heights$height <- as.numeric(CLMA_heights$height)

# clma ramet heights (lengths)
summary(CLMA_height_mod <- lmer(height ~ treatment*bout + (1|unique_id), data =
CLMA_heights)) # removed tree bc no effect
```

```
(clma_height_emmeans <- emmeans(CLMA_height_mod, pairwise~treatment*bout,
adjust= "Tukey"))

# PIGR - - - - - - - - - - - - - - - -
PIGR_stem_hts <- read.csv("~/Documents/UGA /Grad Project/biomass
data/PIGR_stem_ht_csv.csv")
PIGR_stem_hts <- na.omit(PIGR_stem_hts)
PIGR_stem_hts$bout <- as.factor(PIGR_stem_hts$bout)
PIGR_stem_hts$main_stem_height <- as.numeric(PIGR_stem_hts$main_stem_height)

PIGR_height_mod <- lmer(log(main_stem_height) ~ treatment*bout + (1|unique_ind), data
= PIGR_stem_hts) # removed tree bc no effect
summary(PIGR_height_mod)
emmeans(PIGR_height_mod, pairwise~treatment*bout, adjust= "Tukey")
```
